# Barriers and facilitators to developing an equity dashboard to identify disparities in kidney transplant access: a mixed methods study

**DOI:** 10.3389/frtra.2026.1906784

**Published:** 2026-08-18

**Authors:** Rachel Forbes, Marissa C. Kuo, Marianna D. Frazee, James L. Rogers, Praveen Vimalathas, Hannah L. Brooks, Elisa J. Gordon

**Affiliations:** 1 Department of Surgery, Vanderbilt Health, Nashville, TN, United States; 2 Institute of Global Health, Vanderbilt Health, Nashville, TN, United States; 3 Department of Surgery and Center for Biomedical Ethics and Society, Vanderbilt Health, Nashville, TN, United States

**Keywords:** ethics, healthcare disparities, implementation science, multidisciplinary team, patient selection committee, qualitative research, quality assurance and performance improvement, social drivers of health

## Abstract

**Introduction:**

While kidney transplant disparities are well-documented, little is known about disparities in the evaluation process after referral. Electronic dashboards interfacing with electronic health records can disaggregate patient data to identify potential avenues for reducing disparities. This cross-sectional study assessed contextual and design factors that would inform implementation of an ‘Equity Dashboard’ at one transplant center.

**Methods:**

Clinicians, implementation science experts, and patients were interviewed. Qualitative data were analyzed using thematic analysis guided by the Consolidated Framework for Implementation Research. Quantitative data comprised primarily descriptive survey data collected during interviews.

**Results:**

Twenty clinicians/experts and 23 patients participated. Perceived facilitators included: Inner Setting constructs of mission alignment, culture, tension for change, compatibility, and structural characteristics; and Individual Characteristics construct of high-level leaders. Dashboard alignment was expected with institutional commitment to patient-centered care, center workflow, and leadership support. Perceived barriers included: Intervention Characteristics construct of costs; and Inner Setting constructs of access to knowledge and information, information technology infrastructure, and work infrastructure. Financial costs were expected to support information technology development, staff training and time, and interventions arising from dashboard findings.

**Conclusions:**

Our findings suggest that perceived barriers were largely modifiable while perceived facilitators were immutable and integral to the institution, which would facilitate dashboard implementation. Future research will evaluate dashboard implementation on patients’ transplant access.

## 1 Introduction

Disparities in access to kidney transplantation pervade the transplant evaluation process (1–4). Racial and ethnic minorities, women, and patients of lower socioeconomic status and in rural areas are less likely to be referred, evaluated, and added to the waiting list for organ transplantation (5, 6). Greater attention has focused on examining how structural factors may affect transplant access including social drivers of health (SDOH) – the conditions in which people are born, live, and age that affect health functioning and quality-of-life outcomes (4, 5, 7).

Disparities may arise at many points in access to transplantation from referral, evaluation completion, waitlisting, and transplant receipt. Identifying precisely how and when SDOH affect patients’ access to transplantation during the post-referral evaluation process can reveal avenues for intervention at the transplant center level. Commonly identified barriers to screening for SDOH in other clinical contexts include lack of: provider time to ask about SDOH, available resources to address social needs, and training in responding to identified needs (8–10); while commonly identified facilitators include: leadership support, staff buy-in, and availability of a standardized screening approach (9, 11, 12). Yet little is known about barriers and facilitators to screening for SDOH in the transplant context.

Transplant centers are the optimal setting to identify disparities in patients’ access to transplantation. Strategies to identify and reduce SDOH-driven inequities include patient navigation programs, community partnerships, and redesigning care processes to reduce structural barriers. While these strategies can meaningfully improve access and equity, many require personnel, financial costs, or workflow re-design, limiting scalability and sustainability (7, 13, 14).

Alternatively, a comparatively low-cost and feasible strategy to screen for SDOH-based inequities in transplantation entails using a dashboard whereby transplant team members (i.e., clinicians, administrators) monitor patients’ progression throughout the transplant evaluation process. A dashboard (i.e., a visual summary of prespecified datapoints) could leverage existing data infrastructure to systematically identify whether disparities exist by SDOH, monitor known disparities over time, and inform design of targeted interventions. As with any organizational unit, transplant centers must assess disaggregated patient-level data by SDOH variables at different phases of the evaluation process to illuminate where bottlenecks occur and for whom, and establish institutional initiatives toward achieving health equity (15). However, to properly analyze disaggregated patient data and ascertain the existence of potential disparities, healthcare organizations must develop and integrate systems into clinical care (16). Because transplant teams do not routinely discuss equity matters, integrating discussion about equity into routine transplant practice as an easily refreshable visualized report would make the dashboard valuable. Dashboards can provide a scalable, resource-efficient foundation for identifying where inequities occur less expensively than social-support interventions (14).

Across healthcare settings, dashboards are commonly used to monitor and display institutionally agreed upon benchmarks, support root-cause analyses of problems identified through analysis of disaggregated data, and monitor the effects of changes in policies and processes (17–19). Dashboards are typically integrated into information technology (IT) systems, and interface with the electronic health record (EHR) to enable automated, real-time data entry and data review (20). Numerous dashboards designed to reduce health disparities (“equity dashboards”) have been developed for maternal mortality, pediatric healthcare, and emergency medicine (17, 21, 22). Dashboards in the healthcare setting can reduce costs and improve patient satisfaction in the inpatient setting, but outcomes depend on context-specific factors (14).

While transplant programs routinely collect center-level data per Organ Procurement and Transplantation Network requirements, it is unknown to what extent programs systematically review their data to improve equity in access to transplantation. No studies have evaluated contextual factors affecting the design and implementation of a dashboard in the transplant evaluation context. This paper aimed to inform dashboard development, procedures for deployment, and assess barriers and facilitators to the future implementation of a dashboard designed to increase equity in patients’ access to transplantation.

## 2 Materials and methods

### 2.1 Study design

This prospective, cross-sectional single-center study involved a mixed-methods concurrent triangulation design (23). Qualitative data were collected through semi-structured interviews, and quantitative data were collected via surveys administered during interviews to gain a broader understanding of the topic by obtaining complementary insights. Mixed methods can enable the identification of commonalities or discrepancies, which can enhance the validity of results, while leveraging the strengths of qualitative and quantitative approaches (23). The study was approved by the Vanderbilt University Medical Center Institutional Review Board (#31435). Electronic written informed consent was obtained from individuals prior to data collection. The Consolidated Criteria for Reporting Qualitative Research was used to ensure quality reporting (24).

### 2.2 Theoretical framework

The Consolidated Framework for Implementation Research 2.0 (CFIR) is an evidence-based determinant framework used to assess contextual factors influencing intervention implementation in healthcare and other contexts (25). The CFIR is comprised of 38 constructs across five domains, including Innovation Characteristics, Individual Characteristics, Inner Setting, Outer Setting, and Process, which guided the clinician interview on perceived barriers and facilitators to intervention implementation (26). Accordingly, Innovation Characteristics referred to the Dashboard, Individual characteristics referred to people involved in Dashboard creation and use, Inner Setting referred to the hospital system, Outer Setting referred to the surrounding community and State, and Process referred to strategies used to implement the innovation.

### 2.3 Participants and recruitment

#### 2.3.1 Clinicians and implementation science experts

Eligible individuals included kidney transplant program members (e.g., physicians, nurses, administrative leadership, social workers) and implementation science experts identified through Vanderbilt Health's Center for Clinical Quality and Implementation Research. Clinicians and experts were recruited by email.

#### 2.3.2 Patients

Eligible individuals included adult (18 + years), English-speaking patients who were referred to Vanderbilt Health for kidney transplantation, waitlisted for a kidney transplant, or had received a kidney transplant from 1 to 1-2016 to 8-21-2023. We obtained a list of 14,170 potentially eligible patient names for recruitment; after removing duplicates, 10,528 patients remained. We randomized patients in the list, and then contacted three sequential sets of *n* = 100 patients for recruitment using a HIPAA-compliant EHR patient messaging system until reaching our target sample of *n* = 25 or study deadline.

### 2.4 Data collection

#### 2.4.1 Clinicians/experts

Semi-structured interviews were conducted by Teams from 2 to 27-2024 to 4-26-2024. Interviews assessed participants’ perceptions of transplant evaluation steps and SDOH (27) that should be tracked by the dashboard; approaches to measuring and classifying disparities with the dashboard; barriers and facilitators to implementing the dashboard (see Supplementary Material 1); and demographics. The interview guide was informed by the CFIR framework, designed to elicit information relevant to the study aims, and reviewed by research team members for refinement. Interview questions included 55 open-ended and 12 closed-ended questions covering the following domains: intervention characteristics, inner setting, outer setting, implementation process, and characteristics of individuals, equity dashboard variables, transplant evaluation steps, measuring disparities, and quality assurance and performance review. One experienced female research team member (EJG) conducted the clinician/expert interviews; she had a professional relationship with some clinicians/experts prior to the interviews. As a social scientist researcher uninvolved in the clinical work of the participants, she was able to quickly establish rapport and participants openly disclosed a broad array of perspectives. This relationship had no impact on analysis because all transcripts were de-identified. One female research team member (RF) uninvolved in data collection, leveraged her clinical insights to interpret clinician participants’ responses during analysis. As thematic saturation can be derived from a minimum of 15 interviews (28), we had established an *a priori* plan to stop data collection at *n* = 20 interviews to include a broad range of clinicians while considering study deadlines and funding constraints. Interviews were audio-recorded, transcribed by Teams artificial intelligence, and verified by one female team member (HLB). Interviews lasted an average of 55 min (range: 32–61). Participants were compensated with a $10 e-gift card unless they declined it.

#### 2.4.2 Patients

Semi-structured interviews were conducted by phone using Teams from 2-27-2024 to 4-26-2024. Interviews assessed patients’ perceptions of the transplant evaluation steps and SDOH variables (27) that should be tracked by the dashboard, and demographic characteristics including health literacy (29). The interview guide was informed by the disparities literature (5), and was reviewed by the research team for refinement. Interviews included 7 open-ended and 17 closed-ended questions covering the following topics: transplant evaluation steps, social determinants of health, and measuring disparities (see Supplementary Material 2). One female research team member (HLB) conducted the interviews and did not have a relationship with participants prior to the interviews. Interviews were audio-recorded, transcribed by Teams artificial intelligence, and verified by HLB. Interviews lasted an average of 33 min (range: 14–69). Participants were compensated with a $40 e-gift card.

### 2.5 Qualitative analysis

Transcripts were analyzed using deductive coding methods (30) guided by the CFIR framework (31). Codes comprised CFIR-based constructs regarding clinicians’ and subject matter experts’ perceived barriers and facilitators to transplant equity dashboard implementation. Research team members (HB, MK, MF, JR, PV) underwent structured training in coding by reading qualitative analysis articles, reviewing guidelines EJG developed on coding and segmenting transcript text, and by practicing coding as a team, to build the codebook. The code definitions within the codebook were refined iteratively and saturation was operationalized as when little or no changes were made to the codebook (28). Each transcript was independently coded by at least two research team members and inter-rater reliability was assessed iteratively until reaching a Cohen's kappa coefficient of >0.80. Coding discrepancies were resolved through consensus discussions among coders and EJG, during which code definitions and applications were reviewed and clarified.

Code summaries were produced for each code by research team members, reviewed and edited by the senior author, and revised by team members. Constructs were categorized as barriers or facilitators to equity dashboard implementation independently by two co-authors and senior author [EJG], and disagreements were settled via discussion between authors.

## 3 Results

### 3.1 Participant characteristics

#### 3.1.1 Clinicians/experts

Twenty clinicians/experts participated out of 32 invited (62.5% participation rate). Most participants were female (85.0%), White (65.0%) or Black (20.0%), and between the ages of 31–40 years (45.0%) (Table 1).

**Table 1:** Clinician and implementation science expert demographic and clinical characteristics, *N* = 20.

Variable	*n* (%)
Age	
31–40 years	9 (45.0)
41–50 years	7 (35.0)
51–60 years	3 (15.0)
61–70 years	1 (5.0)
Gender	
Female	17 (85.0)
Male	3 (15.0)
Ethnicity	
Not Hispanic or Latino	18 (90.0)
Hispanic or Latino	2 (10.0)
Race	
White	13 (65.0)
Black or African American	4 (20.0)
Asian	2 (10.0)
Multi-Racial	1 (5.0)
Years in Practice	
≤ 5 years	2 (10.0)
6–10 years	3 (15.0)
11–20 years	10 (50.0)
21–30 years	5 (25.0)
Professional Role	
Clinical Coordinator (NP, PA, RN)	6 (30.0)
Social Worker	3 (15.0)
Transplant Surgeon	2 (10.0)
Transplant Nephrologist	2 (10.0)
Quality Assurance	2 (10.0)
Administrator	2 (10.0)
Research	2 (10.0)
Financial Coordinator	1 (5.0)
Professional Degrees	
MD/DO	6 (30.0)
APN/RN	5 (25.0)
SW/LSW/MSW	3 (15.0)
MPH/MS/MSPH/MBA	3 (15.0)
Other	2 (10.0)
PhD	1 (5.0)

#### 3.1.2 Patients

Of 283 patients invited to participate, 233 did not reply, 10 replied as not interested, and 40 replied as interested. Of the 40 expressing interest in participating, 7 responded after the recruitment deadline. Of the 32 who expressed interest during the recruitment period, 23 completed an interview, 8 did not reply to recruitment messages, and 1 was no longer eligible. The participation rate for patients expressing interest during the recruitment period was 72% (*n* = 23/32), and for all sent the recruitment message was 8.1% (*n* = 23/282). Most participants were female (60.9%), White (61.0%), and between the ages of 61–70 years (26.1%) (Table 2).

**Table 2:** Patients’ demographic and clinical characteristics, *N* = 23.

Variable	*n* (%)
Age, years, mean [SD] (range)	
31–40 years	5 (21.7)
41–50 years	5 (21.7)
51–60 years	2 (8.7)
61–70 years	6 (26.1)
70+	5 (21.7)
Gender	
Female	14 (60.9)
Male	9 (39.1)
Ethnicity	
Not Hispanic or Latino	21 (91.3)
Hispanic or Latino	1 (4.3)
Prefer not to disclose	1 (4.3)
Race	
White	14 (61)
Black or African American	3 (13.0)
Other	3 (13.0)
Prefer not to disclose	2 (8.7)
American Indian or Alaska Native	1 (4.3)
Marital Status	
Married/Domestic partner/Civil union	14 (60.9)
Separated or divorced	4 (17.4)
Never married/single	3 (13.0)
Widowed	1 (4.3)
Education	
High school graduate	1 (4.3)
Some college	4 (17.4)
College graduate	13 (56.5)
Post graduate degree (MA, PhD, MD, DO, etc.)	4 (17.4)
Employment Status	
Employed full-time	4 (17.4)
Employed part-time	2 (8.7)
Not employed	12 (52.2)
Disabled	4 (17.4)
Income	
Less than $15,000	3 (13.0)
Between $15,000 and $34,999	6 (26.1)
Between $35,000 and $54,999	2 (8.7)
Between $55,000 and $74,999	2 (8.7)
Between $75,000 and $94,999	2 (8.7)
More than $95,000	7 (30.4)
Primary Health Insurance	
Private health insurance	14 (60.9)
Medicaid or Medicare	6 (26.1)
Other	2 (8.7)
Health Literacy	
Never/rarely	21 (95.7)
Sometimes/often/always	1 (4.3)
Health Status	
Excellent	0 (0)
Very Good	8 (34.8)
Good	6 (26.1)
Fair	7 (30.4)
Poor	1 (4.3)
Cause of End-Stage Kidney Disease	
Polycystic Kidney Disease	6 (26.1)
Hypertension	2 (8.7)
Glomerulonephritis	1 (4.3)
Doctors do not know	1 (4.3)
Other/Multiple causes	12 (52.2)
Time on Dialysis	
<1 year	6 (26.1)
1–3 years	2 (8.7)
4–6 years	5 (21.7)
Never	9 (39.1)
Received Kidney Transplant	
Yes	15 (65.2)
No	7 (30.4)

### 3.2 Barriers and facilitators to transplant equity dashboard implementation

We organized clinicians’ perceived barriers and facilitators to equity dashboard implementation by CFIR domains of Individual Characteristics, Innovation Characteristics, and Inner Setting. Clinicians’ representative illustrative quotations are presented below.

#### 3.2.1 Individual characteristics

*High level leaders* emerged as a facilitator. Participants anticipated that the transplant center's and organization's leadership would provide strong project buy-in because leadership had already demonstrated strong support for endeavors promoting equitable care. As one participant stated:

“I think there's a good foundation already for support in this area. Equities become a huge component in transplant. We've already pushed out some of that work trying to identify some areas for opportunity. So I think that that's something I think you already have a baseline foundation of support from the leadership.” (#7008)

Such buy-in would indicate alignment of the project with broader institutional goals and a readiness for institutional change. One respondent said that leadership would have a vested interest in supporting the dashboard for advancing leadership's goal of increasing transplant volumes by identifying and overcoming barriers to transplantation, as expressed in the following:

“I think there's definitely an interest and effort to increase our transplant volumes… and then the ways we do that is also by identifying barriers to transplant for whatever reasons that would be. And so I do think there would, there should be support from our leadership to try to help our patients get to transplant and ways that we can do that…. I feel like there is at least buy-in or support from leadership to help facilitate this.” (#7019)

Respondents recognized the need for sustained and proactive leadership involvement to ensure the dashboard's effective integration and sustainability within clinical workflows. Together, these responses suggest that participants viewed leadership support not simply as administrative endorsement, but as a prerequisite for securing organizational commitment, allocating resources, and sustaining dashboard implementation over time.

#### 3.2.2 Innovation characteristics

*Costs* were the most commonly reported barrier. Participants identified several potential costs relating to dashboard implementation, the primary ones being staff time and the long-term interventions arising from dashboard findings but reckoned that the costs would be “negligible.” Staffing costs included identifying individuals to spearhead dashboard management, maintaining staff responsible for interpreting data, and onboarding ancillary help from other departments. For example, one participant said:

“In a resource limited environment, understanding how we would support an additional data entry I think would be one thing that we would definitely need to think through. And then database maintenance, you know, who’s responsible for developing the database and maintaining it.” (#7022)

Others identified the costs to build, set up, and maintain the dashboard. Participants anticipated additional costs to purchase new processing/data systems for dashboard development. At the time of the interviews, most respondents perceived that the initial outlay of costs may be greater, but thereafter, minor investment would be required for a refreshable dashboard, as one commented:

“It’s gonna cost money, but I think once the original design is sort of established, it should be pretty automatic is sort of how I've seen them work. … if this is something that the institution values…, I don't imagine the cost to be astronomical for something like this” (#7015)

Although costs were identified as a potential barrier, participants generally distinguished between the initial investment required to develop the dashboard and the relatively limited resources anticipated for its long-term maintenance. This distinction suggests that concerns centered more on implementation than sustainability.

#### 3.2.3 Inner setting

Inner Setting facilitators included: *mission alignment*, *culture, structural characteristics, compatibility,* and *tension for change.*

*Mission Alignment* was a prominent facilitator as many respondents emphasized this point. Respondents perceived that the equity dashboard directly aligned with the transplant center's commitment, purpose, and goals, as evidenced by the transplant teams’ values of equity and inclusion, improvement and quality, and access to transplant and patient care. Specifically, respondents recognized how the dashboard could: a) promote equity and inclusion by fostering non-discrimination and ensuring equal access to transplant services for all patients; b) support improvement and quality by enhancing the transplant center's effectiveness in addressing identified inequities; and c) facilitate access to transplant and patient care by providing transplants to as many eligible patients as possible. As one participant shared:

“I think the dashboard highly aligned with the mission of the transplant center, which is to be able to provide … lifesaving organs to anyone that can benefit from them. And so we know that there are large groups of patient populations that don't have access to transplant, and we need to fix that. And I think this would help to fix that.” (#7016)

Through these mechanisms, respondents anticipated that the dashboard could advance the transplant center's ability to waitlist and transplant patients, patients’ quality of life, and patients’ post-transplant care and outcomes. The transplant center's goals also aligned with the organizational pillars of diversity, equity, and inclusion, equity and access, and personalized healthcare, which reinforced the dashboard's perceived relevance. One participant summed up these points well:

“I think it would directly align with the transplant center’s mission, and not only within the transplant center, but just within the Vanderbilt Health organization altogether. Our organization’s goal is to make healthcare personalized, and we have our patient and family promise. And then within transplant specifically, the goal is to be able to provide a new quality of life for our patients and to be able to provide a means to move forward in the setting of the chronic illnesses that a lot of our patients have…. And so I think that this dashboard would definitely … meet that vision and to meet those goals.” (#7011)

Across interviews, participants consistently linked the dashboard to the transplant center's broader mission of equitable, patient-centered care. Rather than viewing the dashboard as an additional administrative task, many described it as a mechanism to operationalize existing institutional priorities.

Another commonly recognized facilitator was transplant center *culture*. Participants placed strong emphasis on maintaining a culture that values diverse backgrounds and perspectives, which would be critical to the dashboard's success. One participant succinctly described the culture in the following:

“I do think there’s some general shared beliefs, like obviously, the people who work in the transplant center believe in transplant for improved quality of life and equal access to transplant…” (#7001)

Participants emphasized the importance of establishing and maintaining shared values among the team because a unified vision and common goals would facilitate collaboration and enhance the future effectiveness of the dashboard after implementation. As one participant explained:

“I think the culture of our transplant center is what makes us so successful, because I think everybody at every level as a whole is really passionate about the work that we do and is truly invested in trying to help these patients and get them through the process and ultimately to get them transplanted…. I think something that you see in here all the time is that people are going above and beyond…. And so I think particularly if there’s findings that show inequities that we need to address, I think everybody will step up and do whatever’s needed to do to fix that, because that’s the culture we have here. We, the team, is just filled with a lot of passion about what they do.” (#7007)

Participants highlighted the need to provide all faculty and staff with training in equity and using the dashboard, which would facilitate understanding the necessity of the dashboard and its role in enhancing equitable care.

The transplant center's *structural characteristics* comprised another facilitator. Participants viewed the transplant program's large size as both an asset and a logistical challenge. Respondents equated the large transplant volume of patient referrals and the diverse patient population to providing sufficient data to identify disparities, which would help to establish the dashboard. Similarly, the large volume of available clinical staff who evaluate patients would facilitate dashboard implementation. However, one participant detailed some logistical considerations of the large program for dashboard development and implementation:

“Our kidney transplant program is very big. We have a lot of staff members in that program. A lot of several different key roles. And, we have a lot of patients and so the throughput is pretty quick. … it’s kind of a churn there.. It’s a machine just so because of the high volumes there. And so I think that’s just something to think through when you have such high volumes moving through that program and the ability to think about how would we collect this data, how would we make it efficient, how would we minimize what we needed to collect again because just the volume there is so high.” (#7022)

These considerations underscored the importance of designing efficient dashboard data collection and reporting processes.

Several also noted how the strength of the transplant program, in terms of its maturity and prominent leadership, would foster implementation. How leadership strength would contribute to dashboard deployment was conveyed clearly in the following:

“I think we have a very mature leadership structure. We have a mechanism for these ideas to be proposed to the transplant center and then we have mature meetings and schedules, conferences that we can introduce the concept of, do the rollout.” (#7016)

Another facilitator was the perceived *compatibility* of the dashboard with the transplant program. Participants anticipated that the dashboard would fit well within the transplant program's workflows, systems, and processes. Respondents believed that the infrastructure was already in place to integrate the dashboard into daily functions, given the data-centered approach to transplant practice oversight, as one clinician explained:

“So … this is a very data-driven center. We have a lot of opportunities for touch points for data, and outside of the QAPI meetings, there’s also directors’ meetings and their staff meetings and that kind of thing. So I think our teams are really familiar with looking at data and that kind of being a part of their utilizing that information to make clinical actions. So I think that that’s a natural process that'll fit nicely into what we've already structured.” (#7008)

For example, many identified the Quality Assurance and Performance Improvement (QAPI) meetings for discussing dashboard findings. While participants identified a variety of routine, frequently occurring meetings in which the dashboard could be reviewed, the QAPI was perceived as an optimal venue:

“So I think it fits in well in QAPIs especially because that’s where we're, like, really looking at the program as a whole. What’s going on? What’s on the horizon? So I think it would fit in well.” (#7002)

*Tension for change* was commonly identified as a facilitator. Respondents recognized a modest need to change how the transplant team addressed disparities arising in patient evaluation. First, participants remarked that some team members lacked a clear understanding of the extent to which the transplant team delivered equitable care. Consequently, several reported being unaware of current patient inequities but envisioned that the dashboard would help address this, as one participant related:

“I think there’s a great need. I think we sort of know that there’s a problem, but don't know how to tackle it or the magnitude of the problem. And I think that having a real time tool to give us some visual analytics would be very helpful at the time of decision making across the spectrum from referral to eval to transplant” (#7010)

Second, respondents reported how well-intentioned policies may have inadvertently disqualified underserved patients from transplantation, leading to inequitable care. For example, participants referred to the policy of identifying patients as ineligible for transplantation if patients missed more than 5 treatments, and how not examining why patients were missing treatments may contribute to the inequity. One participant explained how the dashboard is needed because it could be useful in addressing such situations:

“I think there’s a good need for it. …[S]ometimes we just end up turning patients down because they don't go through the testing or they don't complete it in a timely fashion… I don't think we really investigate too strongly into or we might identify some reasons, but we don't, I guess, really try to intervene or do anything to try to help correct it.” (#7019)

Participants surmised that not comparing different groups of patients (i.e., underserved versus affluent) would lead to inequitable care. Respondents expressed motivation to resolve this matter and expected the dashboard could be used toward this end. Lastly, participants stated that the transplant team sought to provide more equitable care, motivating respondents to change the way the transplant team was currently evaluating patients. Collectively, these responses reflect recognition that disparities likely exist within the current evaluation process but are not routinely measured or discussed. Participants viewed the dashboard as a practical tool for making these inequities visible and informing targeted quality improvement efforts.

Inner setting barriers included: *access to knowledge and information*, *IT infrastructure*, and *work infrastructure*.

A commonly identified barrier was *access to knowledge and information*. Participants repeatedly mentioned that dashboard implementation would require resources including time and training, particularly up front, but not over time, as one participant explained:

“Short term? Yes. Long term? No, I think once it’s one of those things that’s in place, they're pretty good about if something needs to be built through Epic to gather information, then our team is also made getting that done. Then it just becomes who owns it, who reviews it. But I don't think long-term would require a lot of resources.” (#7003)

Participants recognized that a fundamental understanding of technology would be necessary, although they anticipated the training to access the dashboard would likely not be extensive. Respondents noted that the availability of time could pose a challenge regarding IT support, data collection, and the initial build and management of the dashboard, particularly at a busy academic hospital. One participant highlighted extensive logistical concerns that would need to be addressed for dashboard development and maintenance:

“I think possibly IT resources. If we find out that there’s data that we're not tracking or not getting that we don't have a system in place to get that information, and then also just having staff buy-in, and making sure that you have people that are committed to getting you the information that you need, and then teaching those staff members how to get that information and what they're looking for” (#7013)

Respondents acknowledged how existing resources, such as the transplant team and IT department, could be utilized, but leveraging these resources would require coordination. Participants also anticipated that training in clinical use of the dashboard would be required to effectively integrate it into practice.

A widely recognized barrier was *information technology infrastructure*. Respondents emphasized the importance of securing health IT's support and identifying specific team members with expertise in health science informatics. IT buy-in would help ensure that necessary resources were available to build the dashboard with little to no cost or need for additional staffing, as one participant conveyed:

“I think that we have a lot of people on our team that are good with those things and that can create it, however we need it to be created. So, I think we have the resources at that technology wise to make it happen.” (#7021)

IT support would facilitate the integration of the dashboard within the existing EHR, which would simplify dashboard use and enhance accuracy of data collection. Some respondents anticipated challenges in creating the physical appearance of the dashboard within the EHR. Others also stressed the need for training staff in the use of the dashboard in order for the dashboard to be used appropriately:

“As long as there is some adequate training, I think it would be fine. Epic can be very confusing so adding something else to it,… as long as we have the training, I think it'll be fine, but sometimes it does feel like things are being implemented that everybody hasn't received the same amount of training on and then if no one’s, so it’s not being utilized to the best of his abilities, because people aren't doing the same thing.” (#7018)

Another commonly identified barrier was *work infrastructure*. Participants reported concerns about how dashboard implementation would add more responsibilities to an already overextended workforce. As one participant explained, additional responsibilities introduced by the dashboard could receive varied responses depending on how difficult the new work would be:

“We all get comfortable on a certain workflow, the way we work, but then when you add in something else, that sort of like throws somewhat, I wanna say, a monkey wrench, into how you work. So some people might push back because they'll be like ‘oh man, … we'll have to do something else..’ But I think if it’s not challenging, if it’s user friendly, then it would probably go over well.” (#7005)

Respondents identified potential challenges of integrating the dashboard into existing workflows including: set up (i.e., pre-determining dashboard content, defining data elements, and visualization standards), methods of data collection, development of mechanisms for maintenance, determining how findings would be used, and disseminating dashboard data. Participants expressed concerns about how dashboard data would be interpreted and acted upon within the transplant workflow, particularly within the high-volume, multidisciplinary clinical setting. The introduction of the dashboard into the workflow raised a number of questions for participants:

“But the collecting the information part, and gathering it, is what I would be worried about. Who would be in charge of that? And would we have the staff to support that? Because I feel like right now, everyone is, they have quite a bit of … responsibility. So would there be other? I'm sure there wouldn't be other people. … I mean this would be added on to someone else’s responsibility list.” (#7012)

The potential for variation in dashboard data collection or interpretation would threaten dashboard consistency and reliability.

### 3.3 Design and implementation considerations

Clinician and patient participants offered recommendations regarding the future development and deployment of the transplant equity dashboard.

#### 3.3.1 Transplant evaluation phases

Of the 15 clinicians/experts and 23 patients surveyed, all agreed to include 6 of the 8 evaluation phases in the dashboard (Table 3). These phases included the total number of patients referred for transplantation, percentage of referred patients who were waitlisted, received a kidney transplant (overall), received a deceased donor kidney transplant, and received a living donor kidney transplant. Some clinicians considered the time from referral to the first evaluation as an unreliable metric because insurance may cause delays outside of the patient's control. A non-significant trend emerged whereby more clinicians than patients recommended including the number of days it took patients to complete evaluation before the selection committee meeting (100% clinicians versus 82.6% patients, *p* = 0.083). Patient participants suggested also tracking pre-referral phases (i.e., the severity of patients’ kidney failure, adherence to dialysis, and access to living donors), reasons for delays during the evaluation process, and post-transplant outcomes (i.e., transplant success and length of recovery).

**Table 3:** Patient and clinician ratings of evaluation steps for inclusion in the dashboard.

Evaluation steps selected for inclusion in the equity dashboard	% Patients (*N* = 23)		% Clinicians (*N* = 15)	
	Yes	No*	Yes	No*
Number of patients who were referred for transplantation	100	0	100	0
Of referred patients, the number of patients who attended the first in-person evaluation	91.3	8.7	93.3	6.7
Number of days it took patients from referral to attending the first in-person evaluation	73.9	26.1	86.7	13.3
Of referred patients, the number of patients who finished all tests and exams	95.7	4.3	100	0
Number of days it took patients from referral to finishing all tests and exams	82.6	17.4	100	0
Of referred patients, the number of patients who were reviewed by the Kidney-Pancreas (KP) committee	95.7	4.3	93.3	6.7
Number of days it took patients from referral to being reviewed by the KP committee	95.7	4.3	93.3	6.7
Of referred patients, the number of patients who were placed on the waiting list	100	0	100	0
Number of days it took patients from referral to being placed on the waiting list	100	0	100	0
Of referred patients, the number of patients who received a kidney transplant (overall)	100	0	100	0
Number of days it took patients from referral to receiving a kidney transplant	95.7	4.3	93.3	6.7
Of referred patients, the number of patients who received a deceased donor kidney transplant	100	0	100	0
Number of days it took patients from referral to receiving a deceased donor kidney transplant	95.7	4.3	93.3	6.7
Of referred patients, the number of patients who received a living donor kidney transplant	100	0	100	0
Number of days it took patients from referral to receiving a living donor kidney transplant	95.7	4.3	93.3	6.7

N*, number of patients or clinicians asked the question; “Possibly” included as Yes; “I don't know” included as No.

#### 3.3.2 SDOH variables

All clinicians/experts agreed to include 8 of the 25 SDOH listed, while patients did not agree on including SDOH variables in the dashboard (Table 4). Leading SDOH variables with the greatest agreement between patients and clinicians included: patient age, access to healthcare services, and healthcare communications. Participants suggested also tracking patients’ ability to pay for hotels and transportation so that the transplant team can financially assist these patients.

**Table 4:** Patient and clinician ratings of SDOH for inclusion in the dashboard.

SDOH variables	Patients (*N* = 23)			Clinicians & experts (*N* = 20)		
	*N* ^a^	Yes (%)	No (%)	*N* ^a^	Yes (%)	No (%)
Access to health services, including dental and prescriptions (ease, affordability, quality)	23	87.0	13.0	19	100	0
Citizenship/immigration status	23	69.6	30.4	18	100	0
Employment and income, especially degree of security	23	47.8	52.2	19	100	0
English proficiency	23	69.6	30.4	19	100	0
Ethnicity and race	23	65.2	34.8	19	100	0
Health literacy/numeracy	23	82.6	17.4	19	100	0
Healthcare communications	23	87.0	13.0	19	100	0
Neighborhood (poverty rate, educational attainment of neighbors, safety, characteristics that influence physical activity)	23	60.9	39.1	19	100	0
Access to the internet and health technology	23	87.0	13.0	19	94.7	5.3
Current age	23	91.3	8.7	19	94.7	5.3
Religion and spirituality	23	43.5	56.5	19	94.7	5.3
Individual educational attainment	23	52.2	47.8	19	94.7	5.3
Disability status	23	82.6	17.4	18	94.4	5.6
Housing (quality, location, affordability)	23	56.5	43.5	18	94.4	5.6
Gender identity	23	39.1	60.9	19	89.5	10.5
Marital/Family status (access to support)	23	78.3	21.7	18	88.9	11.1
Physical activity	23	78.3	21.7	18	88.9	11.1
Food (ease of access, quality, affordability)	23	78.3	21.7	19	84.2	15.8
Racial/ethnic discrimination experienced outside of healthcare settings	23	87.0	13.0	19	78.9	21.1
Racial/ethnic discrimination experienced in healthcare settings	23	34.8	65.2	19	78.9	21.1
Current address	23	73.9	26.1	18	77.8	22.2
Community environmental conditions (air quality, exposure to toxins)	22	63.6	36.4	19	68.4	31.6
Sex assigned at birth	23	69.6	30.4	19	68.4	31.6
History of incarceration (personal, family)	23	26.1	73.9	19	42.1	57.9
Birthplace	23	39.1	60.9	19	36.8	63.2

*N*^a^: *N*, asked question.

#### 3.3.3 Integration with QAPI meetings and communication strategies

All clinician/expert respondents emphasized the importance of engaging the entire multidisciplinary team when discussing dashboard data at QAPI meetings because “it takes a collective effort to address these things.” Many recommended presenting the dashboard at quarterly meetings and providing an explanation about the rationale for and relevance of dashboard implementation to the transplant program. Respondents suggested that QAPI meetings establish a “safe space” when discussing dashboard findings and set expectations that not all identified disparities can be acted upon. Dashboard accessibility was especially important for ensuring transparency and eliciting input from all disciplines, thereby enhancing the discussion about patients’ transplant eligibility.

## 4 Discussion

### 4.1 Summary of findings

**Figure 1:**
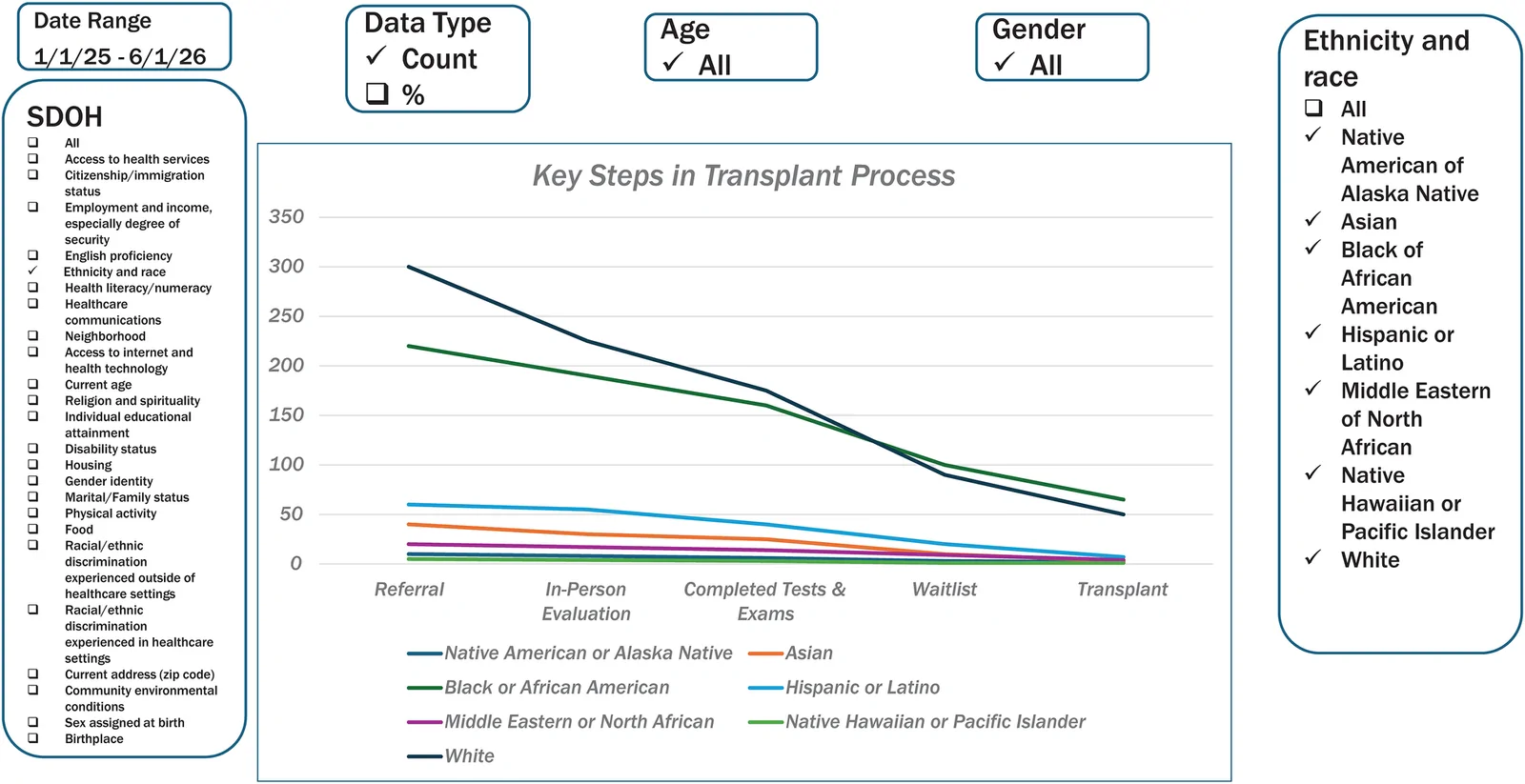
SDOH [ethnicity and race] by key steps in transplant process.

**Figure 2:**
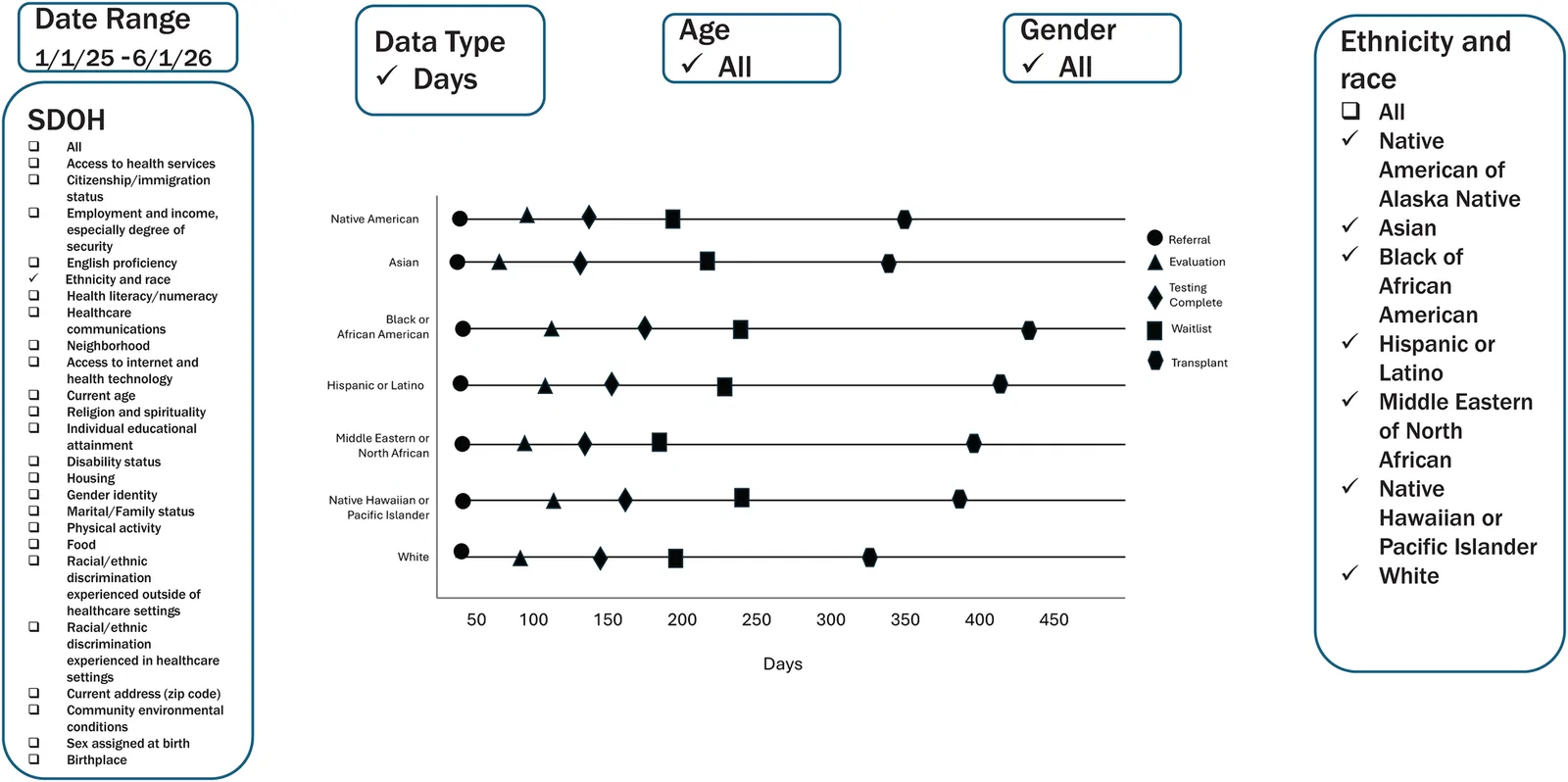
SDOH [ethnicity and race] by time (days) in transplant process.

Our mixed-methods study provides novel insights into kidney transplant clinicians’ and patients’ perceptions of transplant Equity Dashboard development and implementation into transplant clinical practice. We found high levels of support for deploying the dashboard into routine clinical practice to track and identify disparities in the evaluation process for potential intervention during our study period. Based on participants’ feedback variables to be included in the Equity Dashboard, we developed two dashboard mock-ups (Figures 1, 2).

We identified noteworthy facilitators to implementing a transplant Equity Dashboard, including the Inner Setting constructs of *tension for change*, *culture,* and *mission alignment;* and the Individuals construct of *high-level leaders.* Overall, dashboard implementation would be strongly facilitated by a commitment from transplant team members and institutional leaders to ensure equitable access to transplantation.

### 4.2 Comparison with existing literature

Our findings coincide with research outside transplantation demonstrating how aligning dashboard goals with existing institutional and participant values helps facilitate dashboard use (32). Empirical research in business management describes the design of dashboards to create mission and goal alignment and drive innovation implementation (33–35). Our study suggests a reverse causal relationship: rather than the dashboard creating mission alignment, existing institutional values were expected to facilitate dashboard adoption. Facilitator constructs appear to be integral personal and institutional values, which may explain this observation.

We also identified barriers to implementing an Equity Dashboard in the transplant context, namely, the Inner Setting constructs of: financial *costs*, *access to knowledge and information*, and *IT* and *work infrastructure*. Participants’ concerns about costs of dashboard development, staff time, and interventions have been echoed in other research. For example, a systematic review of EHR dashboards tracking medication-related outcomes found that cost-effectiveness was a priority guiding dashboard design (36). At the time of our study, there was considerable institutional and national support for equity research. Our study participants believed these costs would *not* pose prohibitive barriers and transplant center infrastructure could support dashboard design and implementation given that institutional leaders perceived the topic as important. The case for cost-effectiveness to justify funding dashboard development would be the anticipated increase in number of waitlisted and transplanted patients. Once developed, the dashboard would require minimal maintenance and could potentially become cost-effective.

Participants emphasized the need for staff training to use and analyze dashboard data (*access to knowledge and information)*, but did not anticipate these concerns would pose a prohibitive barrier. Similarly, a systematic review of disparity dashboards highlighted the importance of considering intended users and their information needs when designing the dashboard to minimize the impact on existing workflows and resources (17). Studies have advocated for clarity on the intended dashboard use and user and recommended creating different user interfaces for different roles to maximize engagement (17), which may decrease time needed for staff training.

Study participants' reported need for *IT infrastructure* support for dashboard development and deployment aligns with studies evaluating dashboard implementation. Research demonstrates how IT support can ensure the dashboard is user-friendly for different roles and useful in clinical practice. Transparent data sharing, ease of access to IT support, scheduled meetings with IT experts, regular trainings, and regular review of dashboard results as a multidisciplinary team have all been shown to improve dashboard use and usability. Involving key stakeholders in the dashboard design process and convening frequent sessions for users in alleviating these concerns have predicted dashboard usability (36–38).

Our findings advance existing literature by identifying contextual factors uniquely affecting dashboard implementation and design in the transplant context. In 2021, the Organ Procurement and Transplantation Network developed an ‘Equity in Access to Transplant’ dashboard with the goal of assessing the impact of SDOH on waitlisting and transplantation (39). However, the Organ Procurement and Transplantation Network's dashboard does not assess the impact of SDOH from referral throughout the evaluation process, which our proposed Equity Dashboard aims to do.

### 4.3 Implications for research and practice

Monitoring patients’ progress throughout the transplant evaluation process by SDOH is valuable for identifying precisely where in the process and which particular social drivers disproportionately contribute to disparities. Our study participants regarded most evaluation steps and SDOH as worthy of inclusion in the Equity Dashboard. There is no consensus on the number of variables that should be included in dashboards in the healthcare setting. Dashboards vary widely and commonly include numerous indicators (17, 40, 41). A scoping review recommended careful selection of key performance indicators to avoid viewer overload or fatigue (41).

Clinicians expressed a preference for including more SDOH in the dashboard than patients, which may reflect clinicians’ commitment to upholding institutional and transplant team values of equity, quality improvement, and access to transplant care. While clinicians preferred including access to healthcare, education access, and neighborhood and built environment variables, patients preferred including social and community context variables. This finding underscores the importance of engaging patients in dashboard design to ensure responsiveness to their needs, which can improve transparency and trust (42). Requiring 100% agreement between the transplant team and patients on SDOH variables and evaluation steps may not be feasible. Establishing a threshold cut-off for agreement, however, requires balancing the desire for specificity and equity with the need for feasibility and practicality.

The clinician-identified barriers and facilitators to Equity Dashboard implementation are actionable because they provide practical guidance to the institution and transplant team on preparations for Dashboard deployment. Similarly, the clinician- and patient-generated quantitative data are valuable for informing the prioritization of relevant SDOH variables and evaluation steps for Dashboard inclusion.

Our next steps entail building the Dashboard and conducting a modified Delphi method including clinicians and patients to refine Dashboard variables and integration into existing workflows.

We anticipate granting special consideration to patient-prioritized variables identified through the present study in the development of the Equity Dashboard.

The convergence between qualitative themes and quantitative survey responses strengthens confidence that the proposed dashboard content reflects stakeholder priorities rather than isolated opinions. Areas of disagreement, particularly where clinicians favored inclusion of more variables than patients, provide important direction for the planned modified Delphi method, where stakeholder perspectives can be reconciled through consensus building.

In instances of disagreement between patients and clinicians, we will undertake several steps including: leverage empirical data to validate their perspectives, conduct further interviews to better understand the rationale for inclusion or exclusion of the variable from the Equity Dashboard, and create dashboard views that have patient-centric variables. We do not anticipate designing the dashboard to be patient-facing; the transplant center may not be equipped to address upstream, nonmodifiable risk factors contributing to disparities, which could lead patients to misinterpret the data. Our plan is to integrate the dashboard into the EHR, which will be updated in real time based on discrete variables which we will start to proactively include. We plan to use the dashboard during routine, quarterly QAPI meetings whereby the full transplant team will review and monitor the dashboard results. We expect that a team of clinicians (e.g., a transplant surgeon, transplant nephrologist, social workers, and nurse coordinators) will lead the Equity Dashboard review process during the meetings and act on the findings. We will prioritize intervening in all phases of the evaluation process in order to support access to transplantation. Identified disparities will lead to interventions based on faculty expertise in the topic, institutional resources to support quality improvement projects, and by securing leadership and transplant-team wide consensus on intervening on the topic. Despite the federal administration's elimination of support for equity-related research, enthusiasm expressed by the institution for the Equity Dashboard has all but diminished, yet initiatives have been paused. A renaissance of institutional support for equity research is anticipated in future years.

### 4.4 Strengths and limitations

This study has strengths including investigating a novel application of equity dashboards for reducing health disparities, particularly in the pre-waitlist transplant context. A notable strength of this study is the deliberate inclusion of patient perspectives alongside clinician input to guide the selection of Equity Dashboard variables, ensuring that dashboard development reflects the priorities of the populations it intends to serve. Our qualitative findings provide a rich description of barriers and facilitators to dashboard implementation, which will inform the design and implementation of our Equity Dashboard. Our mixed-methods approach provided confirmatory validation of quantitative and qualitative findings.

This study has some limitations. As a single center study, the barriers and facilitators we identified may not be generalizable to other transplant programs. However, many factors we identified have been reported in other clinical contexts, suggesting external applicability. Replicating this study at other transplant programs may reveal the transferability of study findings. A limitation is that the Dashboard has not yet been developed, which may have made responding to questions about the Dashboard challenging for clinicians and patients. Interview guides were not pilot-tested due to limited resources, which may have diminished question face and content validity. Clinician and expert participants’ responses may have been shaped by social-desirability bias. While we did not interview IT representatives, interviewed clinicians had backgrounds in dashboard reporting using Epic and quality improvement. Low patient participation rates were attributed to the opt-in approach involving the patient portal for recruitment; other studies using this recruitment strategy have documented similar or lower participation rates (i.e., 2.9%–7.4%) (43, 44). However, when calculating the patient participation rates based on those who replied to our initial Epic recruitment message as a denominator, the 46% rate is much more reasonable. Despite institutional requirements known at the time of the study that restricted patient recruitment through the portal, we recognize that recruitment through the portal may have introduced a selection bias in favor of including patients who are more digitally connected, more educated, more engaged, or more comfortable using electronic communication. Racial/ethnic disparities in using the EHR for recruitment are unlikely given that Vanderbilt Health has a very high patient portal usage rate. We did not include non-English speakers, which may under-estimate barriers faced by non-English-speaking patients, and may limit generalizability of results. The absence of non-English speakers may underestimate the barriers reported by the very populations whom the Dashboard aims to track. Most study participants were White and highly educated patients, which may have limited the range of barriers reported. The sample size is small for quantitative analyses.

### 4.5 Conclusions

In conclusion, our findings suggest that perceived barriers to implementation of an Equity Dashboard were largely modifiable while perceived facilitators were immutable and integral to the core values of the participants and the institution. Our findings will inform future dashboard development and implementation. We anticipate that addressing barriers and facilitators would optimally support the implementation of the Equity Dashboard in the transplant context.

## Data Availability

The original contributions presented in the study are included in the article/Supplementary Material, further inquiries can be directed to the corresponding author.
