# Supplementary material for: Barriers and facilitators to developing an equity dashboard to identify disparities in kidney transplant access: a mixed methods study
Source: Front Transplant. 2026 Aug 18;5:1906784. doi: 10.3389/frtra.2026.1906784 (PMC13529691; doi:10.3389/frtra.2026.1906784)
Supplement: Supplementary file 1 [file Supplementaryfile1.pdf]

Supplemental File 1. Provider / Expert Interviews

Rachel Forbes, MD, MBA
Elisa Gordon, PhD, MPH
"Development of an Equity Dashboard and Processes for Clinical Integration"

Equity Dashboard: Transplant Team Interview Guide (Version 4)

<u>Introduction</u>:

I'll refer to the intervention as the "Equity Dashboard".

Our goal is for the Equity Dashboard to reduce disparities for patients seeking a kidney transplant.

The Equity Dashboard has 2 parts:

1. System:

- We are developing a Dashboard that will disaggregate data on patients undergoing evaluation for kidney transplantation.
- Disaggregating patient data is important to better understand how social determinants of health contribute to disparities in patients' ability to finish the evaluation process.
- This Dashboard will then show us where we need to direct our attention in order to change practices or policies at the institutional level to mitigate disparities.

2. Processes:

- We are developing procedures for the Quality Improve Healthcare Research and Quality (AHRQ) defines disparities as any differences among populations that are statistically significant and differ from the reference group by at least 10 percent.6 Oftenment team to review the Dashboard, to evaluate the data, determine what disparities to act upon, and determine how to respond to disparities.
- Through this process, the Equity Dashboard will raise awareness of disparities at the Transplant Center level with the goal of responding in ways that mitigate inequities for our patients.

The goal of this interview is to ask your opinion on how we could integrate the Equity Dashboard into QAPI meetings.

Do you have any questions before we begin?

Date of IRB Approval: 12/01/2023
Institutional Review Board
VANDERBILT

CFIR Domain	CFIR Construct	Interview Questions	Definition
**Intervention Characteristics**			
	Adaptability	1. What kinds of changes do you think you will need to make to the **Equity Dashboard intervention** so it will work well in the Transplant Center?<br><br>2. Do you expect the **Equity Dashboard** to be adaptable/modifiable in these ways?	The degree to which an intervention can be adapted, tailored, refined, or reinvented to meet local needs.
	Complexity	1. How complicated is the **Equity Dashboard** to deliver?<br><br>2. What challenges do you anticipate with deploying the **Equity Dashboard**?<br><br>3. What challenges do you anticipate with integrating the **Equity Dashboard** into your kidney transplant program and QI process?	Perceived difficulty of the intervention, reflected by duration, scope, radicalness, disruptiveness, centrality, and intricacy and number of steps required to implement.
	Cost	1. What costs (including financial and opportunity costs) do you anticipate the **Equity Dashboard** having on our transplant program during the rest of this research study?<br><br>2. What costs (including financial and opportunity costs) do you anticipate the **Equity Dashboard** having on our transplant program after this research study ends?<br>4.	Costs of the intervention and costs associated with implementing the intervention including investment, supply, and opportunity costs.
	Implementation Readiness<br><br>Leadership Engagement	1. Is there buy-in from organizational leadership to support the **Equity Dashboard** intervention during this research study?<br><br>2. What kind of support have you needed to keep the **Equity Dashboard** running during this research study?<br><br>3. What buy-in from organizational leadership do you anticipate needing to support the **Equity Dashboard** after this research study ends? What kind of support have you needed for this study?<br><br>5. 4. What kind of support do you anticipate needing to keep the **Equity Dashboard** running after this research study ends?	Commitment, involvement, and accountability of leaders and managers with the implementation.

Date of IRB Approval: 12/01/2023
Institutional Review Board
VANDERBILT

**Inner Setting**			
	Culture	1. What are some of the shared values and beliefs shared within the Transplant Center that may affect (positively or negatively) the implementation of the **Equity Dashboard**?	There are shared values, beliefs, and norms across the Inner Setting.<br>Four archetypical organizational cultures arise: 1) **team culture** (high internal focus with high flexibility (aka personal)); 2) **hierarchical culture** (high internal focus with high control (aka formalized and structured)); 3) **entrepreneurial culture** (high external focus with high flexibility (aka dynamic and entrepreneurial)); and 4) **rational culture** (high external focus with high control (aka production oriented))<br>There are shared values, beliefs, and norms about the inherent equal worth and value of all human beings.
	Structural characteristics	2. How will the infrastructure of the kidney transplant program (social architecture, age, maturity, size, or physical layout) affect the implementation of the **Equity Dashboard**?<br><br>3. What kinds of infrastructure changes will need to be made to accommodate the **Equity Dashboard**?	The social architecture, age, maturity, and size of an organization.
	2. Information Technology Infrastructure	4. How will the Transplant Center's information technology systems of electronic documentation, and data storage, management, reporting, and analysis affect the functional performance of the **Equity Dashboard**?	Technological systems for tele-communication, electronic documentation, and data storage, management, reporting, and analysis support functional performance of the Inner Setting.
	3. Work Infrastructure	5. How might the Transplant Center's organization of tasks and responsibilities within and between individuals and teams, and general staffing levels, affect the functional performance of the **Equity Dashboard**?	Organization of tasks and responsibilities within and between individuals and teams, and general staffing levels, support functional performance of the Inner Setting.
	<u>Organizational Climate:</u><br><br>Tension for Change	I'd like you to reflect on the how you and other transplant stakeholders at VUMC are currently identifying and reducing disparities for under-represented kidney patients in getting access to transplantation.<br><br>1. How great is the need to change the way the transplant team is address disparities in completing evaluation, waitlisting, and receiving a transplant among under-represented patients?	The degree to which stakeholders perceive the current situation as intolerable or needing change.

Date of IRB Approval: 12/01/2023
Institutional Review Board
VANDERBILT

	Compatibility	1. To what extent does the **Equity Dashboard** intervention fit with our transplant program's norms, workflows, systems? How does it not fit?	The degree of tangible fit between meaning and values attached to the intervention by involved individuals, how those align with individuals' own norms, values, and perceived risks and needs, and how the intervention fits with existing workflows and systems.
	Relative Priority	1. To what extent is the **Equity Dashboard** intervention a priority for our transplant program compared to other priorities?<br><br>2. What makes the **Equity Dashboard** intervention (more or less) important than other priorities?	Individuals' shared perception of the importance of the implementation within the organization.
	Available Resources	1. Is the **Equity Dashboard** going to require more resources (like time, physical space, and training) than our transplant program currently has available?<br><br>2. What resources will the **Equity Dashboard** require from our transplant program?<br><br>3. How well will our transplant program be able to accommodate those resource needs?	The level of resources dedicated for implementation and on-going operations, including money, training, education, physical space, and time.
	Mission Alignment	1. To what extent will implementing the **Equity Dashboard** be in line with the Transplant Center's overarching commitment, purpose, or goals?	Implementing and delivering the innovation is in line with the overarching commitment, purpose, or goals in the Inner Setting.
**Outer Setting**			
	Market Forces [Peer Pressure]	1. To what extent will the **Equity Dashboard** give Vanderbilt's transplant program an advantage compared to other transplant programs in our area?	Mimetic or competitive pressure to implement an intervention; typically because most or other key peer or competing organizations have already implemented or are in a bid for a competitive edge.
	Local Attitudes	2. What attitudes (beliefs, values) in the community (e.g., VUMC, Nashville, or professional) can encourage VUMC to support delivery of the **Equity Dashboard**?	Sociocultural values (e.g., shared responsibility in helping recipients) and beliefs (e.g., convictions about the worthiness of recipients) encourage the Outer Setting to support implementation and/or delivery of the innovation.
	Local Conditions	*3.* What economic, environmental, political, and/or technological conditions enable the VUMC to support implementation of the **Equity Dashboard?**	Economic, environmental, political, and/or technological conditions enable the Outer Setting to support

Date of IRB Approval: 12/01/2023
Institutional Review Board
VANDERBILT

			implementation and/or delivery of the innovation.
	3. Performance-Measurement Pressure	4. To what extent will quality or benchmarking metrics or established service goals drive implementation of the **Equity Dashboard?**	Quality or benchmarking metrics or established service goals drive implementation and/or delivery of the innovation.
**Process**	***Implementation Process:*** The activities and strategies used to implement the innovation.		
	Assessing Needs:<br>B1. Innovation Deliverers	1. What priorities, preferences, and needs of people delivering the intervention will guide implementation of the **Equity Dashboard?**	Collect information about the priorities, preferences, and needs of deliverers to guide implementation and delivery of the innovation.
	Assessing Needs:<br>B2. Innovation Recipients	2. What are the priorities, preferences, and needs of recipients of the intervention that will guide implementation of the **Equity Dashboard?**	Collect information about the priorities, preferences, and needs of recipients to guide implementation and delivery of the innovation.
	C. Assessing Context	3. What are the barriers and facilitators to implementing the **Equity Dashboard?**	Collect information to identify and appraise barriers and facilitators to implementation and delivery of the innovation.
	E. Tailoring Strategies	4. What strategies will be used to address barriers, leverage facilitators, and fit the **Equity Dashboard** into the QI context?	Choose and operationalize implementation strategies to address barriers, leverage facilitators, and fit context.
	Doing<br>[Executing]	5. What factors will affect our transplant program's ability to implement the **Equity Dashboard** intervention?<br><br>6. Will your transplant program implement the **Equity Dashboard** intervention according to the implementation plan that we are setting up? Or in some other way?	Carrying out or accomplishing the implementation according to plan.<br>2.0: Implement in small phases, steps or cycles of change that cumulatively build, before scaling up more broadly with continued optimization until [Innovation] becomes routine.

**Characteristics of Individuals**			
	Capability	What interpersonal competence, knowledge, and skills do you have to engage in implementing or to support the implementation of the **Equity Dashboard?**<br>a) in terms of working on the mechanics of creating and using a dashboard (generically speaking)?<br>b) in terms of responding to what we find when using the Equity Dashboard?	The individual(s) has interpersonal competence, knowledge, and skills to fulfill Role.
	Motivation	How committed are you in implementing or supporting the implementation of the **Equity Dashboard?**	The individual(s) is committed to fulfilling Role.

Date of IRB Approval: 12/01/2023
Institutional Review Board
VANDERBILT

Equity Dashboard Development (System)
This section is about creating the Equity Dashboard.

1. What information should be presented in the Equity Dashboard?

2. Which variables should be displayed graphically?

3. The Table below lists all social determinants of health. I'll review each one and let me know which SDOH variables should be tracked in the Equity Dashboard.

Table 1. Social determinants of health variables [2]	
	Respondent Recommended Variables (*Mark 'x' for each variable preferred*)
**Core**	
Access to health services and technology	
Age	
Annual family income	
Biological sex assigned at birth	
Birthplace	
Citizenship/immigration status	
Current address	
Current age	
Current employment status	
Disability status	
Discrimination and Disparate health care quality	
Educational attainment-individual	
Employment	
English proficiency	
Ethnicity and race	
Food insecurity	
Gender identity	
Health insurance coverage	
Health literacy/numeracy	
Job insecurity	
Marital status	
Occupational prestige	
Sexual orientation	
Zip code-derived variables	
**Individual**	
Wealth	
Job insecurity	
Access to health IT	
Discrimination	
Disparate health care quality	
Health numeracy	
**Structural**	
Air Quality Index	
Concentrated Poverty	
Educational Attainment - Community	
Environmental Justice	
Food Swamp	
Percent Unionized for Non-Agricultural Labor Force	
Race/Ethnic Residential Segregation (ACS)	
Race/Ethnic Residential Segregation (unbiased)	
Race/Ethnic Residential Segregation (census)	
Social Vulnerability	

1. After patients get referred to the transplant program, getting tested for a transplant happens over a few steps.

Some groups of patients make it through the testing process more than other groups of patients.

We are thinking about tracking which patient groups make it and don't make it through each step so that the transplant team can first identify disparities and then work on fixing the disparities.

Now, I'm going to show you different steps in this process. For each step, I'd like to ask you whether we should track which patients make it through that particular step.

Transplant Testing Steps	Yes	No
Number of patients who were referred for transplantation		
Of referred patients, the number of patients who attended the first in-person evaluation		
Number of days it took patients from referral to attending the first in-person evaluation		
Of referred patients, the number of patients who finished all tests and exams		
Number of days it took patients from referral to finishing all tests and exams		
Of referred patients, the number of patients who were reviewed by the Kidney-Pancreas (KP) committee		
Number of days it took patients from referral to being reviewed by the KP committee		
Of referred patients, the number of patients who were placed on the waiting list		
Number of days it took patients from referral to being placed on the waiting list		
Of referred patients, the number of patients who received a kidney transplant (overall)		
Number of days it took patients from referral to being receiving a kidney transplant		
Of referred patients, the number of patients who received a deceased donor kidney transplant		
Number of days it took patients from referral to being receiving a deceased donor kidney transplant		
Of referred patients, the number of patients who received a living donor kidney transplant		
Number of days it took patients from referral to being receiving a living donor kidney transplant		

2. Can you think of any other steps in the transplant testing process that should be measured?

3. Sometimes differences between population groups can vary from month to month or quarter to quarter. For example, in one month we might find that women receive more transplants than men, and then in the next month, women receive the same amount of transplants as men. For how long should the population group differences last before recognizing a disparity and taking action to fix it?

Quality Assurance and Performance Improvement (QAPI) Meetings (Process)
This next set of questions are your suggested approaches to using the Equity Dashboard during the QAPI meetings to increase equity in the Transplant Center.

Historical Approaches
1. Does the Transplant Center typically analyze data to figure out if a disparity has occurred?
___ Yes
___ No

If Yes, how did the Transplant Center operationalize (define/measure) "disparity"? How could the Center tell that a disparity exists?

If Yes, How has the Transplant Center responded to the disparity?

Measuring Disparities
Sometimes different patient populations have different health, based on their gender, race, income, and other factors. It is unclear when differences between populations become disparities between populations.

1. How should we measure disparities?

2. Should we use the proportion of all referred by demographic category? (e.g., of all women referred, xx% initiated in-person evaluation)

Here are some ways to think about disparities:
- Disparities are unjust or unfair.
- One definition (AHRQ) is that the populations need to differ statistically by at least 10% compared to the dominant group.

3. What are your first impressions?

4. Which percentage difference would you recommend using to prompt Center initiatives to reduce disparities?

5. OK, now let's imagine, for example, that:
- Of all female patients referred to the transplant center, half of them or 50% (or 50 out of 100) were waitlisted.
- Of all male patients referred to the transplant center, most of them or 60% (or 60 out of 100) were waitlisted.

5a. This is a 10% difference between female and male patients. What do you think about this? Should the Transplant team try to fix this or let it go?

5b. Is 10% difference enough to justify taking action on fixing the disparity?

6. What if there was a 2% difference (like 50% and 52%) – Should the Transplant team try to fix this or let it go?

7. What do you think the smallest difference between groups should be in order for the Transplant team to start fixing it?

QAPI Discussions

1. How can we utilize QAPI meetings to review equity data to meet our agreed upon benchmarks?

2. How do we get buy in to proceed with this process?

3. What established approach do you think we should use to help guide discussion around the data?

4. Should this be a separate meeting outside routine Quarterly QAPI meetings?

3. Who should attend the Equity discussion meeting?

6. How often should the Equity meetings occur?

___ Monthly
___ Quarterly
___ Every 6 months
___ Annually

7. We have proposed several questions to guide discussion during the QAPI meetings. I will say each one aloud. For each question, could you say whether you think that question should be used or should not be used?

	Proposed Questions	Should Use	Should **Not** Use
a.	Why is the disparity occurring?		
b.	Should VUMC act upon this disparity? (e.g., develop a new initiative to address this disparity)		
c.	Do you think the disparity can be reduced by changing clinical practice or VUMC policy?		
d.	What suggestions for improvement are there (what has been done in the literature?)		
e.	Are you willing to devote VUMC resources to reduce the disparity?		
e.	Do you think this disparity can be reduced with the appropriate resources and efforts?		
	Can you suggest of other questions?		
f.			
g.			
h.			
i.			

6. What would drive the Transplant Center to work on reducing a disparity identified by the Equity Dashboard?

7. Do you have any further suggestions?

Date of IRB Approval: 12/01/2023
Institutional Review Board
VANDERBILT

Demographics
Lastly, let's wrap up by going over some demographic questions.

1. What is your current age?
   - ☐ < 30 years
   - ☐ 31-40 years
   - ☐ 41-50 years
   - ☐ 51-60 years
   - ☐ 61-70 years
   - ☐ 71 + years

2. What gender identity do you identify most with?
   - ☐ Male
   - ☐ Female
   - ☐ Non-Binary
   - ☐ Transgender
   - ☐ Prefer not to disclose
   - ☐ Prefer to self-describe: ____________

3. Are you Hispanic or Latino?
   - ☐ Yes, I am Hispanic/Latino
   - ☐ No, I am not Hispanic/Latino

4. What is your race (check all that apply)
   - ☐ Asian
   - ☐ White
   - ☐ Black or African American
   - ☐ Native Hawaiian or Other Pacific Islander
   - ☐ American Indian or Alaska Native
   - ☐ Multiracial
   - ☐ Other: ____________

5. How many years have you been in practice?
   - ☐ ≤ 5 years
   - ☐ 6-10 years
   - ☐ 11-20 years
   - ☐ 21-30 years
   - ☐ 31 + years

6. What is your professional role?
   - ☐ Transplant Surgeon
   - ☐ Transplant Nephrologist
   - ☐ Administrator
   - ☐ Clinical Coordinator (NP, PA, RN)
   - ☐ Medical Record Administrator (PCPC)
   - ☐ Social Worker
   - ☐ Transplant Psychiatry
   - ☐ Transplant Pharmacist
   - ☐ Financial Coordinator
   - ☐ Other: ______________

7. What are your professional degrees? (check all that apply)
   - ☐ MD/DO
   - ☐ PhD
   - ☐ MPH/MS/MSPH/MBA
   - ☐ JD
   - ☐ APN/RN
   - ☐ SW/LSW/MSW
   - ☐ Other (please specify):_____________

Thank you!
