# Supplementary material for: Barriers and facilitators to developing an equity dashboard to identify disparities in kidney transplant access: a mixed methods study
Source: Front Transplant. 2026 Aug 18;5:1906784. doi: 10.3389/frtra.2026.1906784 (PMC13529691; doi:10.3389/frtra.2026.1906784)
Supplement: Supplementary file 2 [file Supplementaryfile2.pdf]

Rachel Forbes, MD, MBA
Elisa Gordon, PhD, MPH
"Development of an Equity Dashboard and Processes for Clinical Integration"

Equity Dashboard: Patient Interview Guide (Version 3)

Introduction:
In this interview, we're going to talk about a new program called the "Equity Dashboard".

Our goal is to use the Equity Dashboard to identify disparities for patients going through testing to get a kidney transplant.

The Equity Dashboard has 2 parts:

1. System:
- Transplant programs in the country routinely look at the total number of patients combined in their center who reach key steps in the transplant evaluation process, like getting waitlisted or receiving a kidney transplant.
- Transplant programs use a dashboard, which is like a dashboard in a car, which visually shows information in graphs and tables.
- We are developing a new Dashboard called an Equity Dashboard.
- The Equity Dashboard is going to show more detailed information of population groups, based on demographic factors like sex, race, urban or rural location of residence.
- For example, our Equity Dashboard could show how many women compared to men get placed on the waitlist.
- This way, the Transplant program can see exactly: a) which patient groups may be experiencing problems in completing the testing process, and b) which demographic factors are related to those problems.
- If differences between patient groups occur, like between women and men, for reasons that are avoidable and unfair, then we can call the difference a 'disparity'.
- Our goal is to fix these disparities so that there is fairness or equity for all patients in getting a kidney transplant.

2. Process:
- We are also setting up a way for the Transplant team to look at the Equity Dashboard.
- We need to figure out a few things:
    - What counts as a disparity for the Transplant team to fix it?
    - Which patient population demographic factors should be included in the Dashboard?
    - How should the Transplant team fix the disparities that arise?

Do you have any questions before we begin?

Date of IRB Approval: 12/01/2023
Institutional Review Board
VANDERBILT

Equity Dashboard Development (System)
This section is about creating the Equity Dashboard. We are interested in hearing what factors you think should be tracked in this Dashboard. The factors are things like patient data such as age, race, sex, etc.

1. After patients get referred to the transplant program, getting tested for a transplant happens over a few steps.

Some groups of patients make it through the testing process more than other groups of patients, based on their sex, age, race, and other variables, which is not fair.

We want to track which patient groups make it and don't make it through each step so that we can identify and help those struggling to get through.

Now, I'm going to show you different steps in the testing process. For each step, I'll ask you if we should track patients who make it through that particular step.

**Transplant Testing Steps**	**Yes**	**No**
Number of patients who were referred for transplantation		
Of referred patients, the number of patients who attended the first in-person evaluation		
Number of days it took patients from referral to attending the first in-person evaluation		
Of referred patients, the number of patients who finished all tests and exams		
Number of days it took patients from referral to finishing all tests and exams		
Of referred patients, the number of patients who were reviewed by the Kidney-Pancreas (KP) committee		
Number of days it took patients from referral to being reviewed by the KP committee		
Of referred patients, the number of patients who were placed on the waiting list		
Number of days it took patients from referral to being placed on the waiting list		
Of referred patients, the number of patients who received a kidney transplant (overall)		
Number of days it took patients from referral to being receiving a kidney transplant		
Of referred patients, the number of patients who received a deceased donor kidney transplant		
Number of days it took patients from referral to being receiving a deceased donor kidney transplant		
Of referred patients, the number of patients who received a living donor kidney transplant		
Number of days it took patients from referral to being receiving a living donor kidney transplant		

2. Can you think of any other steps in the testing process that should be tracked?

3. The Table below shows all the patient demographic factors that we could track in the Equity Dashboard. I'll go through each one and ask if you think we should include the factor in the Dashboard so that we can track it.

Table 1. Social determinants of health variables [2]	
	Respondent Recommended Variables (*Mark "x" if responded wishes to track it*)
**Core**	
Access to health services and technology	
Age	
Annual family income	
Biological sex assigned at birth	
Birthplace	
Citizenship/immigration status	
Current address	
Current age	
Current employment status	
Disability status	
Discrimination and Disparate health care quality	
Educational attainment-individual	
Employment	
English proficiency	
Ethnicity and race	
Food insecurity	
Gender identity	
Health insurance coverage	
Health literacy/numeracy	
Job insecurity	
Marital status	
Occupational prestige	
Sexual orientation	
Zip code-derived variables	
**Individual**	
Wealth	
Job insecurity	
Access to health IT	
Discrimination	
Disparate health care quality	
Health numeracy	
**Structural**	
Air Quality Index	
Concentrated Poverty	
Educational Attainment - Community	
Environmental Justice	
Food Swamp	
Percent Unionized for Non-Agricultural Labor Force	
Race/Ethnic Residential Segregation (ACS)	
Race/Ethnic Residential Segregation (unbiased)	
Race/Ethnic Residential Segregation (census)	
Social Vulnerability	

Using the Equity Dashboard to Increase Equity (Process)
The next set of questions are about how the Transplant Center can use the Equity Dashboard.

Measuring Disparity

Sometimes different patient populations have different health, based on their gender, race, income, and other factors.

It is unclear when differences between populations become disparities between populations.

Here are some ways to think about disparities:
- Disparities are unjust or unfair.
- One definition (AHRQ) is that the populations need to differ statistically by at least 10% compared to the dominant group.

1. There are different ways to measure disparities in transplantation. We're interested in your thoughts about how big a disparity needs to be between groups in order for the Transplant Team to decide to fix it.

What are your first impressions?

2. OK, now let's imagine, for example, that:
- Of all female patients referred to the transplant center, half of them or 50% (or 50 out of 100) were waitlisted.
- Of all male patients referred to the transplant center, most of them or 60% (or 60 out of 100) were waitlisted.

2a. This is a 10% difference between female and male patients. What do you think about this? Should the Transplant team try to fix this or let it go?

2b. Is 10% difference enough to justify taking action on fixing the disparity?

3. What if there was a 2% difference (like 50% and 52%) – Should the Transplant team try to fix this or let it go?

4. What do you think the smallest difference between groups should be in order for the Transplant team to start fixing it?

Transplant Team Solutions
Now, let's talk about possible solutions to fix the disparities.

1. How should the Transplant team fix the disparities?

2. Do you have any further suggestions?

Demographics
Lastly, let's wrap up by going over some demographic questions.

1. What is your current age? __________ years

2. What gender do you identify most with?
- ☐ Male
- ☐ Female
- ☐ Non-Binary
- ☐ Transgender
- ☐ Prefer not to disclose
- ☐ Prefer to self-describe: ____________

3. Are you Hispanic or Latino?
- ☐ Yes, I am Hispanic/Latino
- ☐ No, I am not Hispanic/Latino

4. What is your race (check all that apply):
- ☐ White
- ☐ Black or African American
- ☐ Asian
- ☐ Native Hawaiian or Other Pacific Islander
- ☐ American Indian or Alaska Native
- ☐ Multiracial
- ☐ Other: ____________

5. What is the highest grade or level of formal education you have completed?
- ☐ Less than high school graduate
- ☐ High school graduate
- ☐ Some college
- ☐ College graduate
- ☐ Post graduate degree (MA, PhD, MD, DO, etc.)

6. What is your present marital status?
- ☐ Married/Domestic partner/Civil union
- ☐ Separated or divorced
- ☐ Living with partner
- ☐ Never married/Single
- ☐ Widowed

7. What is your current employment status? *Please check one.*
- ☐ Employed full-time
- ☐ Employed part-time
- ☐ Not employed
- ☐ Student
- ☐ Homemaker
- ☐ Disabled

8. What primary health insurance do you have?
- ☐ Private health insurance (through employer or self-pay)
- ☐ Medicaid/Medicare
- ☐ None
- ☐ Other: ____________________________

Date of IRB Approval: 12/01/2023
Institutional Review Board
VANDERBILT

9. The next question may be a bit sensitive. We ask about income because it is an important factor that can affect a person's decisions about health care. What is your total household income for the past 12 months (you + spouse/partner)?

☐ Less than $15,000
☐ Between $15,000 and $34,999
☐ Between $35,000 and $54,999
☐ Between $55,000 and $74,999
☐ Between $75,000 and $94,999
☐ More than $95,000

10. How often do you need to have someone help you when you read instructions, pamphlets, or other written material from your doctor or pharmacy?

___ Never
___ Rarely
___ Sometimes
___ Often
___ Always

11. How confident are you filling out medical forms by yourself?

___ Extremely
___ Quite a bit
___ Somewhat
___ A little bit
___ Not at all

Clinical Questions

1. In general, would you say your health is …… *State the following categories.*

___ Excellent
___ Very Good
___ Good
___ Fair
___ Poor

2. What is the main cause of your kidney disease (end-stage kidney disease)?

☐ Hypertension
☐ Diabetes
☐ Glomerulonephritis
☐ Polycystic Kidney Disease
☐ Other:_________________
☐ Doctors do not know
☐ Prefer not to answer

3. How long have you been on dialysis? _________ months

4. Have you received a kidney transplant?

___ Yes
___ No

5. Do you have any questions?

___ Yes
___ No

6. May I confirm your email address for emailing your gift card?

EMAIL Address: ____

We will send you your e-gift card shortly for your participation.

Thank you very much!
